# Safety and pharmacokinetic profile of fixed-dose ivermectin with an innovative 18mg tablet in healthy adult volunteers

**DOI:** 10.1371/journal.pntd.0006020

**Published:** 2018-01-18

**Authors:** Jose Muñoz, Maria Rosa Ballester, Rosa Maria Antonijoan, Ignasi Gich, Montse Rodríguez, Enrico Colli, Silvia Gold, Alejandro J. Krolewiecki

**Affiliations:** 1 Barcelona Institute for Global Health, ISGlobal-CRESIB, Universitat de Barcelona. Barcelona, Spain; 2 CIM-Sant Pau. IIB Sant Pau. Institut de Recerca de l’Hospital de la Santa Creu i Sant Pau. Barcelona, Spain; 3 Pharmacology and Therapeutics Department, Universitat Autònoma de Barcelona (UAB), Bellaterra, Spain; 4 ExeltisPharma, Chemo group. Madrid, Spain; 5 Fundacion Mundo Sano, Buenos Aires, Argentina; 6 Instituto de Investigaciones en Enfermedades Tropicales, Universidad Nacional de Salta/CONICET, Oran, Argentina; McGill University, CANADA

## Abstract

Ivermectin is a pivotal drug for the control of onchocerciasis and lymphatic filariasis, which is increasingly identified as a useful drug for the control of other Neglected Tropical Diseases. Its role in the treatment of soil transmitted helminthiasis through improved efficacy against *Trichuris trichiura* in combination with other anthelmintics might accelerate the progress towards breaking transmission. Ivermectin is a derivative of Avermectin B1, and consists of an 80:20 mixture of the equipotent homologous 22,23 dehydro B1a and B1b. Pharmacokinetic characteristics and safety profile of ivermectin allow to explore innovative uses to further expand its utilization through mass drug administration campaigns to improve coverage rates. We conducted a phase I clinical trial with 54 healthy adult volunteers who sequentially received 2 experimental treatments using a new 18 mg ivermectin tablet in a fixed-dose strategy of 18 and 36 mg single dose regimens, compared to the standard, weight based 150–200 μg/kg, regimen. Volunteers were recruited in 3 groups based on body weight. Plasma concentrations of ivermectin were measured through HPLC up to 168 hours post treatment. Safety data showed no significant differences between groups and no serious adverse events: headache was the most frequent adverse event in all treatment groups, none of them severe. Pharmacokinetic parameters showed a half-life between 81 and 91 h in the different treatment groups. When comparing the systemic bioavailability (AUC_0_^t^ and C_max_) of the reference product (WA-ref) with the other two study groups using fixed doses, we observed an overall increase in AUC_0_^t^ and C_max_ for the two experimental treatments of 18 mg and 36 mg. Body mass index (BMI) and weight were associated with t_1/2_ and V/F, probably reflecting the high liposolubility of IVM with longer retention times proportional to the presence of more adipose tissue. Systemic exposure to ivermectin (AUC_0_^t^ or C_max_) was not associated with BMI or weight in our study. These findings contribute to further understand the pharmacokinetic characteristics of ivermectin, highlighting its safety across different dosing regimens. They also correlate with known pharmacokinetic parameters showing stable levels of AUC and C_max_ across a wide range of body weights, which justifies the strategy of fix dosing from a pharmacokinetic perspective.

**Trial registration:**

ClinicalTrials.gov NCT03173742.

## Author summary

Current efforts for the control of poverty-related diseases provide drug treatments through mass drug administration (MDA) as a key component. Ivermectin is an antiparasitary drug which has been used to fight some of these diseases, and millions of treatments have been distributed with a favorable toxicity profile. The dosing strategy of ivermectin is based on weight, which in view of the safety characteristics of ivermectin might not be necessary, while a fix dosing strategy might improve logistics and access to the drug to those who need it. This study was conducted in healthy adult volunteers in which we compared 3 treatment regimens: the weight-based reference standard versus 2 experimental regimens of fix-dose 18 and 36 mg using 18 mg tablets. All 54 volunteers received the 3 treatments sequentially. The results confirmed that the fixed-dose regimen (both 18 mg and 36 mg) are as safe as the standard dosage and could justify the use of fix dosing regimens rather than the current weight based strategy.

## Introduction

The control of Neglected Tropical Diseases (NTDs) has in ivermectin (IVM) the most significant tool among all the drugs used for morbidity control and interruption of transmision. Due to its impact on onchocerciasis and lymphatic filariasis (LF), this macrocyclic lactone has been used in millions of individuals mainly through the Mectizan Donation Program, achieving goals of breaking transmission in several countries and putting those landmark achievements in the horizon of several other countries [1–3]. The very basic approach to the use of IVM consists in its distribution to entire communities through annual or biannual mass drug administration (MDA) campaigns provided its excellent safety profile [4], whose only significant severe adverse reaction has been determined by its use in *Loa loa* infected individuals due to the life-threatening adverse events in this group [5].

The large experience on the use of IVM in the veterinary world, where it was first introduced in 1981 and the growing perception of its capabilities in human disease since its introduction in 1985 has widened its indications to a growing number of infectious diseases. The wide spectrum of action of IVM includes treatment of *Strongyloides stercoralis*, *Gnathostoma spp*, *Mansonella streptocerca* and ectoparasites such as head lice or scabies [1, 6]. Moreover, it has been evaluated in co-administration with albendazol for the treatment of soil transmitted helminthiasis (STH) showing an increased efficacy compared to albendazole stand-alone against *Trichuris trichiura* [7, 8], therefore increasing the feasibility of achieving transmission interruption, as shown in modelling exercises [9]. Due to its alternative mechanism of action, the addition of IVM to a benzimidazole based regimen lowers the threat of emergence of drug resistance [10], as suggested in modelling studies conducted in veterinary medicine [11]. Furthermore, the recent finding that the endectocide effect of IVM reduces the survival of *Anopheles* mosquitoes that feed on an IVM treated person after a single standard oral dose, supports the integration of IVM-based interventions for the control of multiple tropical infectious diseases [12, 13].

Along with its favorable pharmacodynamic aspects, the pharmacokinetic characteristics of IVM also appear to be opening possibilities to expand its use and access. It has rapid oral absorption, high liposolubility and is widely distributed in the body [14]. Following a standard oral dose in healthy humans, IVM reaches peak plasma levels at 3.4 to 5 hours; and plasma half-life has been reported to be 12 to 66 hours [15, 16]. It is metabolized in the liver through the cytochrome P450 system and excreted almost exclusively in feces [14, 17]. Despite its widespread use, there are relatively few studies on the pharmacokinetics of IVM in humans [18] and a full understanding of the relationship between drug levels and activity is also missing, including the mechanisms related to remnant activity beyond time points when significant drug levels are measured, as has been demonstrated in veterinary and vector-borne diseases studies [19, 20]

Regardless of its safety profile and pharmacokinetic features, IVM is prescribed for all its indications in weight (or height) based regimens, which difficult its administration in MDA interventions and introduces the risk of under-dosing [21]. A fixed and high dose regimen which takes advantage of the wide therapeutic index of IVM is an attractive alternative for improving the distribution and therefore potentially increasing coverage rates of treatment campaigns as has been the case for primaquine in the treatment of malaria [22]. Moreover, a safe and efficacious fixed-dose IVM in formulations different than the traditional 3 and 6 mg tablets would be required if co-formulated with other anthelmintics such as albendazole. The aim of this study was to evaluate the safety and pharmacokinetic profile of two fixed doses of IVM using a newly developed 18 mg tablet formulation compared to a standard dosing regimen at 150–200 μg/kg in healthy adult volunteers.

## Methods

### Study design

This study was designed to evaluate the safety and pharmacokinetic profile of 3 dosing regimens of IVM in 54 healthy adult volunteers stratified in 3 weight groups in an open-label, randomized, crossover phase I clinical trial performed under fasting conditions. The study was single dose, three-period, comprising 3 experimental phases of treatment with different doses of IVM. Each experimental period lasted from at least 12 h prior to drug administration to + 168 h post-dose (7 days). The study was carried out at the Centre d’Investigació de Medicaments (CIM-Sant Pau) of the Hospital de la Santa Creu i Sant Pau, in Barcelona, Spain and registered at clinicaltrials.gov (REF NCT03173742).

### Study drugs, doses and dosing regimen

The drugs administered were IVM 18 mg, manufactured by Laboratorios Liconsa S.A., Spain and IVM Revectina 6 mg, manufactured by Abbott Laboratórios do Brasil Ltda, Brazil [23], both as immediate-released tablets.

**Fig 1:**
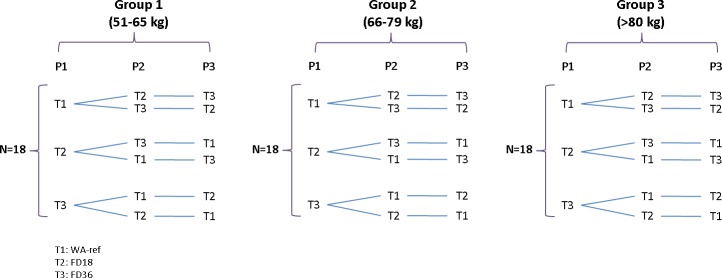
Clinical trial design describing the three different treatment sequences and the wash-out period.

**Fig 2:**
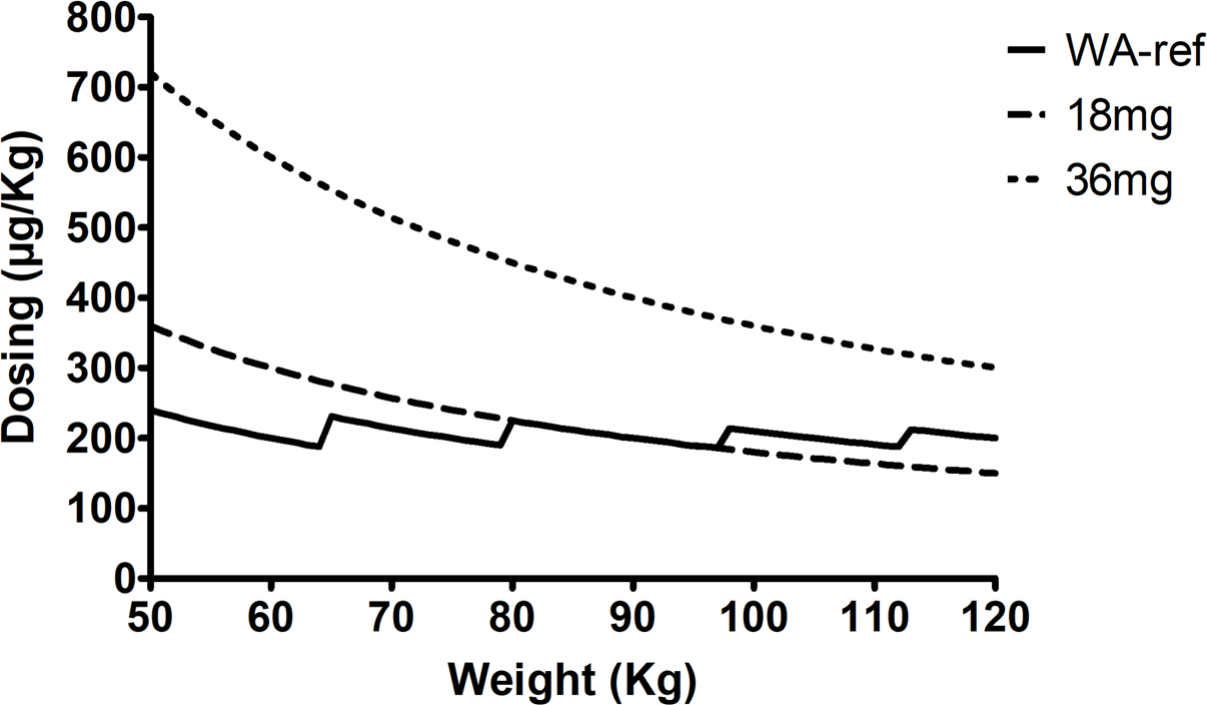
Dosing scheme for the 3 treatment arms with the corresponding weight based exposure for the body weight range of the subjects included in the study. Fixed-dose 18 mg and 36mg with 18 mg tablets and 200μg/kg with 6mg tablets.

Three groups of 18 healthy volunteers with different weights participated in this trial. All participants in each group received three sequential treatments with 240 mL of mineral water. Subjects in Group 1, weighing from 51 to 65 kg, in Group 2, weighing from 66 to 79 kg and in Group 3, weighing ≥ 80 kg received: i) one tablet of IVM 18 mg (FD18), ii) two tablets of IVM 18 mg, 36 mg in total (FD36) and iii) IVM Revectina 200 μg/kg in 6mg tablets (Weight-adjusted reference treatment: WA-ref) using the following sliding scale: 50 to 64.9 kg 2 tabs, 65 to 79.9 kg 2 ½ tabs, 80 to 94.9 kg 3 tabs, 95 to 109.9 kg 3 ½ tabs and 110 to 115 kg 4 tabs. A fourteen-day washout period between each dosing was used (Fig 1). The rationale for the dosing groups was based on the prescription of 2 experimental fixed doses of IVM that provide an amount of drug per kg of body weight that was up to over 700 μg/kg with a minimum of 150 or 300 μg/kg for individuals of up to 120 kg (Fig 2). At the beginning of the study, the subjects were allocated to a randomization number following a procedure of consecutive assignment following a randomization list generated using Windows SPSS software (IBM Corp. Released 2013. IBM SPSS Statistics for Windows, version 22.0. Armonk, NY: IBM Corp) in a balanced way (an equal number of subjects in each treatment sequence).

### Sample size

From the coefficients of variation of the truncated AUC obtained (49.75%) from previous pharmacokinetic studies with ivermectin [15, 16, 24] performed in healthy volunteers, by applying the formula of Sanford Bolton [25] considering an error of type 1 or α of 0.05 and an error of type 2 or β of 0.20, the sample size obtained was 50 volunteers. However, we stratified the volunteers by body weight into 3 groups, (N of 18 volunteers per group, N = 54). Nevertheless, the proposed sample size of 18 volunteers per group was within the range cited in several publications about the pharmacokinetic evaluation of IVM [15, 16, 24] and it was considered adequate for the analysis of the main objective of the study. In order to avoid the possible confounding effect of the sex factor, an imbalance of more than 60%, in the male of female ratio (rounding off to the nearest whole number) was not allowed.

### Subjects and clinical assessments

Participants were male and female subjects aged between 18 and 45 years meeting the inclusion criteria: i) No abnormal findings in medical history and physical examination, ii) Normal laboratory tests (hematology evaluations, blood chemistry and urinalysis), vital signs (systolic and diastolic blood pressure, heart rate and temperature) and ECG record, iii) Not having participated in another clinical trial during the 3 months before starting the current trial and iv) Not having donated blood during the 8 weeks prior to starting the current trial. Female volunteers had to use reliable contraceptive measures not containing hormones. Participants were asked to abstain from drinking alcoholic, xanthine-containing beverages, St John’s Wort, vitamins, herbal remedies and chewing-gum from 48 hours prior to the beginning of the study until study completion. They also agreed to abstain from beverages or food containing grapefruit for 14 days prior to the first study drug administration until study completion. Participants with prior history of alcohol consumption or use or abuse of recreational drugs, consumption of stimulating drinks (> 5 cups of coffee, tea, chocolate or cola drinks per day), smokers or ex-smokers that gave up smoking less than 1 year prior to the study, with history of allergy, idiosyncrasy or serious adverse events and hypersensitivity to drugs or excipients included in drugs, positive serology for hepatitis B, C, or for HIV, and those who took any other medication or medicinal plants in a 15-day period prior to the trial, with history or clinical evidence of chronic diseases, having surgery during the previous 6 months, pregnant or lactating women were excluded. None of the volunteers was at risk of being affected by *Loa loa* or other filarial infections.

During the three treatment periods, urine pregnancy test for female subjects and urine screening for abuse drugs (ethanol, cannabis, cocaine, amphetamines, opiates, benzodiazepines) were performed within the 12 hours before each treatment administration and they were not discharged until +24 h post-medication. Then they returned to the CIM-Sant Pau at +36 h, +48 h, +72 h, +120 h, and +168 h post-dose, for blood extractions.

In each period after fasting overnight for 10 h, subjects received the assigned treatment described above. Fasting continued until +4 h postmedication at which time a standard breakfast was served, followed by a standard lunch at +7 h and a standard dinner at +10 h postmedication. During the experimental phase in each period, volunteers were allowed to drink water *ad libitum* and eat solids from 4 hours after dosing.

### Sample collection and analysis

Nineteen venous samples of 6 mL (2 and 4 mL for IVMB1a and IVMB1b respectively) were collected into EDTA K2 plastic tubes at baseline and at +0.5 h, +1 h, +2 h, +3 h, +3.5 h +4h, +5 h, +6 h, +8 h, +10 h, +12 h, +16 h, +24 h through a cannula placed in the arm of the volunteer and at +36 h, +48 h, +72 h, +120 h, and +168 h post-drug administration by direct venipuncture. Blood samples were centrifuged within 60 minutes after extraction for 10 minutes at 1900 g and at 4°C and the resulting plasma samples were separated into two aliquots of 0.4 and 0.3 mL for IVMB1a and 1.0 and 0.4 mL for IVMB1b respectively that were stored at -20°C ± 5°C until assayed.

Bioanalytical determinations were performed by Anapharm Europe S. L. using a HPLC/MS/MS technique following a full validated method coded 13ANE-2242V and 13ANE-2243V for IVMB1a and IVMB1b respectively according to Guideline on bioanalytical method validation [26], with a limit of quantification of 0.4 ng/mL and 40 pg/mL for IVMB1a and IVMB1b respectively and following the Guide for validation of analytical and bioanalytical methods [27]. Analytical work was performed according to Good Laboratory Practices (GLP).

The calibration curve ranged from 0.40 to 40.00 ng/mL for IVMB1a and from 39.80 to 3980.00 pg/mL for IVMB1b. IVMB1a was extracted by a liquid-liquid procedure with tert-butyl methyl ether, whereas IVMB1b was extracted with a protein precipitation procedure with formic acid 1% prepared in acetonitrile and a subsequent liquid-liquid extraction with dichloromethane. The internal standard for IVMB1a and IVMB1b was doramectin. Within-run accuracy (at 0.40, 1.20, 100.00, 150.00, 200.00 and 2000.00 ng/mL for IVMB1a and at 44.80, 134.40, 2800.00, 4200.00, 5600.00 and 56000.00 pg/mL IVMB1b) ranged from 100.57–109.83% to 103.01–110.37% for IVMB1a and IVMB1b, respectively. Between-run precision was not higher than 5.58% and 10.63% for IVMB1a and IVMB1b, respectively.

### Pharmacokinetic analysis

The Per-Protocol population, (defined as all randomized subjects who met the entry criteria, received all study medication, completed the study and did not present protocol violations) was used for the pharmacokinetic analysis. Pharmacokinetic parameters were estimated from the sum of IVMB1a and IVMB1b plasma concentrations–time data by non-compartmental analysis using Profesional WinNonlin-Pro version 2.1 (Pharsight Corporation, Saint Louis, MO).

Missing samples were treated as non-reportable concentration. In the case of volunteers having plasma concentrations at baseline greater that 5% of C_max_ (in treatment periods 2 and/or 3), we performed a sensitivity analysis by adding or substracting this individual from the analysis in order to evaluate the impact of that volunteer in the pharmacokinetic profile. C_max_ was obtained directly from the plasma concentration–time data. The area under the plasma concentration–time curve (AUC) to the last time with measurable concentration exceeding the limit of quantification (Ct) of the drug (AUC_0_^t^) was estimated by applying log/linear trapezoidal rule. The terminal plasma elimination half-life (t_1/2_) was calculated as t1/2 = 0.693/*ke*, where *ke* represents the first-order elimination rate constant associated with the terminal (log linear) portion of the curve, estimated via linear regression of time versus log concentration. The apparent volume of distribution (V/F) of IVM was calculated as V/F = D/(ke*AUC_0_∞), where D is dose and F is bioavailability and AUC extrapolated to infinity (AUC_0_∞) was determined by adding the extrapolated area (Ct/*ke*) to the AUC_0_^t^. Total oral clearance (Cl/F) was calculated as D/AUC_0_^t^.

### Safety and tolerability assessments

The safety population defined as all randomized subjects who took at least one dose of the study medication was used for safety analyses. Vital signs (systolic/diastolic blood pressure in decubitus and heart rate) were recorded on each treatment day at baseline and at +0.5 h, + 1h, +4 h, +10 h, +24 h, +48 h, +120 h, and +168 h post-dose; hematology evaluations, blood chemistry, and urianalyses were performed at the screening visit in all subjects and at the end of each of the three periods.

Moreover, a complete physical examination and ECG were assessed at the screening visit and at the end of each the three periods. All adverse events observed either by the investigator or reported by the subjects themselves during the clinical study were also recorded and evaluated by the investigator for severity and relationship to the study drug. The severity of each AE was graded according to the following categories: mild, moderate, or severe.

### Ethical statement

The study adhered to the updated Declaration of Helsinki [28] and was conducted according to rules of Good Clinical Practice [29]. Prior to initiation, the study protocol was approved by an independent ethics committee (Clinical Research Ethics Committee of the Hospital de la Santa Creu i Sant Pau, in Barcelona, Spain) and the national competent authority (Spanish Agency for Medicines and Health Care Products, AEMPS, Spain). All subjects provided written informed consent to participate in the study after the nature and purpose of the study was fully explained to them and received stipends for their collaboration.

### Statistical analysis

Descriptive statistics were calculated for all pharmacokinetic parameters as well as a comparison between treatments were performed for all pharmacokinetic parameters. A comparative analysis of bioavailability was applied for the parameters determining exposition in extent [AUC_0_^t^] and rate of absorption [C_max_] without dose correction. Three four-way analysis of variance (ANOVA) for the crossover design were used to assess the effect of dosage, periods, sequences, and subjects-within-sequences on the same parameters. For comparison of the safety and tolerability of IVM between fixed doses (FD18 vs FD36) and between fixed doses and WA-ref adjusted by body weight for each group, an ANOVA and posterior paired analysis with contrast was performed for the parameters obtained in the vital signs, ECG, hematology evaluations, blood chemistry and urianalyses. Additionally, for pooling of all groups, an ANOVA of 2 factors (ANOVA group and treatment arm) and posterior paired analysis with contrast was performed for the same parameters.

The incidence of treatment-emergent AEs (TEAEs) was classified by system organ class and preferred term according to the Medical Dictionary of Regulatory Activities (MedDRA version 20.0), the relationship to the study drug, and the severity for each dose.

Plasma concentrations over 16 ng/ml are generally considered antimosquitocidal for individuals receiving IVM [13]. The minimum, maximum, and median time during which participants had concentrations superior to 16 ng/ml were calculated and compared by means of a Friedman test an a posterior Wilcoxon test, overall and for each weight group.

Additionally, we explored the statistical association of weight and BMI with the main PK parameters (AUC_0_^t^, C_max_, V/F, Cl/F and t_1/2_) by means of an ANCOVA incorporating weight or BMI as a covariates. All the analysis were carried out by mean of Windows SPSS software (IBM Corp. Released 2013. IBM SPSS Statistics for Windows, version 22.0. Armonk, NY: IBM Corp).

## Results

### Subjects

Study population initially included 27 female and 30 male caucasian volunteers without clinical evidence of laboratory, ECG or vital sign abnormalities. None of the randomized subjects were smokers. A description of the most important demographic parameters and other baseline data is shown in Table 1.

**Table 1:** Baseline characteristics of study population (n = 57) in the 3 weight strata. BMI: body mass index.

Group	Variable	N	Mean	SD	Min	Max
Group I (51–65 kg) n = 18	Gender (male/female)	7/11				
	Age (years)	18	26.83	6.09	19	41
	Body weight (kg)	18	58.92	4.01	52	65
	Height (cm)	18	165.57	6.83	156	180
	BMI (kg/m2)	18	21.55	2.15	18.26	25.67
Group II (66–79 kg) n = 19	Gender (male/female)	7/12				
	Age (years)	19	30.32	6.56	22	41
	Body weight (kg)	19	71.46	4.01	66	79.50
	Height (cm)	19	171.89	6.69	159	184
	BMI (kg/m2)	19	24.27	2.08	20.38	26.85
Group III (>80 kg) n = 20	Gender (male/female)	9/11				
	Age (years)	20	29.10	6.74	21	41
	Body weight (kg)	20	87.87	8.46	80	114
	Height (cm)	20	172.85	8.87	158	190
	BMI (kg/m2)	20	29.71	4.99	24.38	45.67

**Fig 3:**
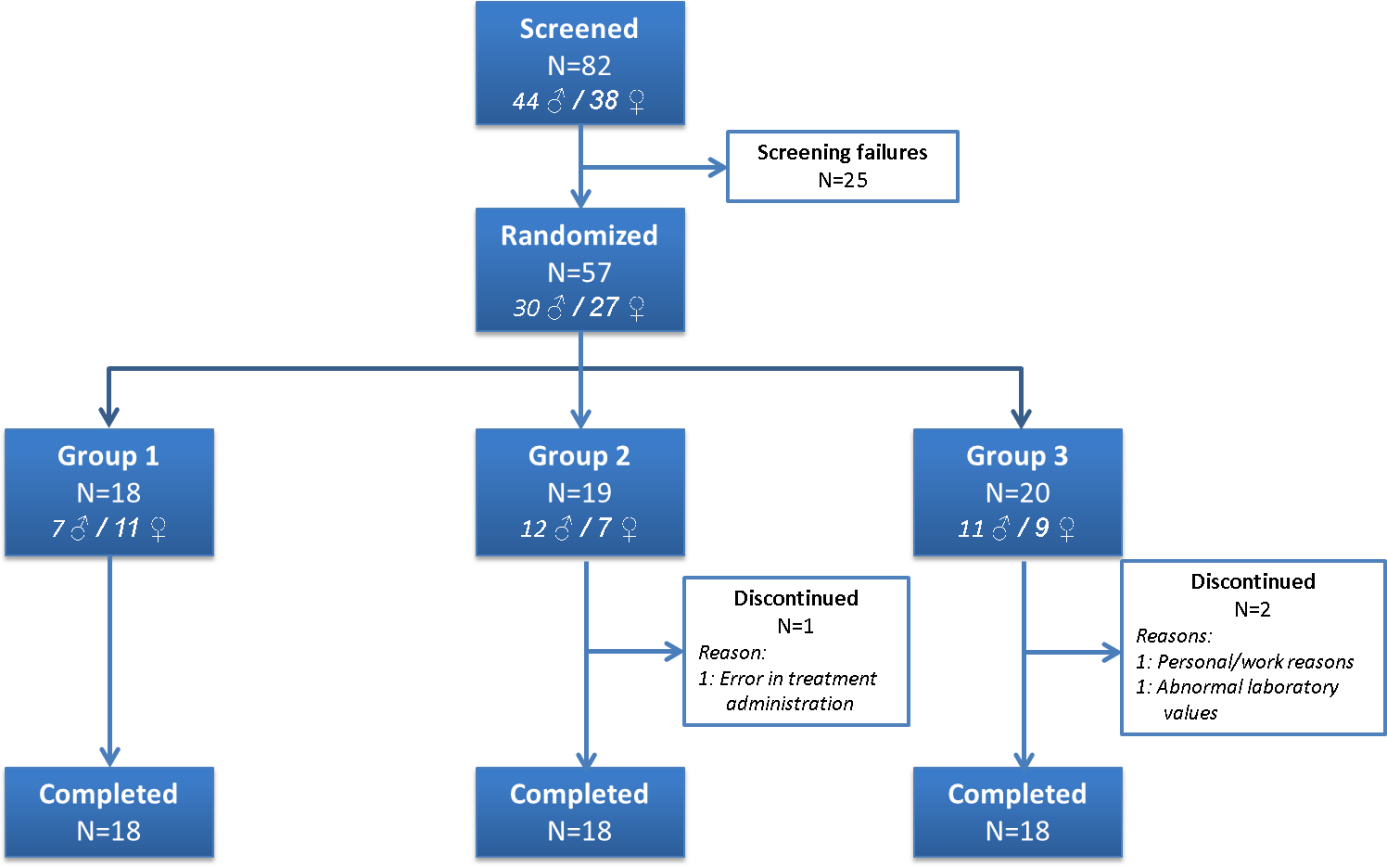
Group 1, subjects weighing from 51 to 65 kg, Group 2 weighing from 66 to 79 kg and Group 3 weighing ≥ 80 kg. Flow diagram of the study.

Fifty-seven volunteers were included in the study and received at least one dose of study drug. After recruitment, one volunteer withdrawn due to personal reasons and two were excluded (one for for a slightly prolonged partial thromboplastin time and the other by protocol deviation in treatment administration) and did not complete the study. A total of 54 volunteers completed the study (Fig 3).

### Safety

All subjects receiving at least one dose of study drug were included in the safety analysis (n = 57). No abnormal result or significant differences were found between biochemistry at baseline and after the administration of IVM in any of the three study arms. A slight decrease in Haemoglobin (Hb) levels was observed after administration of IVM in the three study arms. Hb decreased from 142.80 ± 13.8 g/L at the screening to 137.1 ± 13.55 g/L at the end of the study in the WA-ref (p<0.001), to 136.5 ± 14.51 g/L for FD18 (p<0.001) and to 135.4 ± 12.97 g/L for FD36 (p<0.001). However, no signs or symptoms of anemia were detected in any of the study participants. The main electrocardiographic parameters were not affected by the administration of IVM. Systemic blood pressure measurements were not affected by treatment administration.

A total of 33 treatment emergent adverse events were reported by 22 subjects who received at least one dose of the study medication. Eleven adverse events were reported by 10 subjects after receiving WA-ref, 9 were reported by 9 subjects after receiving FD18 and 13 were reported by 13 subjects after FD36 (Table 2). No significant association was found between the distribution of adverse events and the three treatments arms (p = 0.695). The most frequent adverse event described by study participants was headache (6.02% of the study subjects), followed by dysmenorrhea (5.54%), throat pain (1.80%) and diarrhea (1.80%). Of the 33 adverse events reported, 10 were graded as mild and 23 were graded as moderate. The type and distribution of adverse events by study group are shown in Table 3. Fifteen adverse events were considered possibly related to the investigational products and 18 not related to the investigational products. It was necessary to administer concomitant medication on 14 subjects due to appearance of adverse events (Table 2).

**Table 2:** Distribution of adverse events by frequency and treatment arm. WA-ref: weight adjusted reference group (200 μg/kg), FD18: fixed-dose 18 mg, FD36: fixed-dose 36 mg).

		Treatment				
		Before treatment (N = 57)	WA-ref (N = 54)	FD18 (N = 55)	FD36 (N = 57)	Total (N = 57)
All reported adverse events		1 (1.8%) 1	10 (18.5%) 11	9 (16.4%) 9	13 (22.8%) 13	23(40.4%) 34
Treatment-emergent adverse events (TEAEs)		n.a.	10 (18.5%) 11	9 (16.4%) 9	13 (22.8%) 13	22 (38.6%) 33
Relationship	Study-drug related TEAEs	n.a.	7 (13.0%) 8	2 (3.6%) 2	5 (8.8%) 5	9 (15.8%) 15
	Not study-drug related TEAEs	n.a.	3 (5.6%) 3	7 (12.7%) 7	8 (14.0%) 8	17 (29.8%) 18
Severity	Mild AEs	1 (1.8%) 1	3 (5.5%) 4	2 (3.6%) 2	4 (7.0%) 4	10 (17.5%) 11
	Mild TEAEs	n.a.	3 (5.5%) 4	2 (3.6%) 2	4 (7.0%) 4	9 (15.8%) 10
	Moderate TEAEs	n.a.	7 (13.0%) 7	7 (12.7%) 7	9 (15.8%) 9	23 (40.4%) 23
AEs requiring therapy		1 (1.8%) 1	7 (13.0%) 7	7 (12.7%) 7	7 (12.3%) 7	14 (24.6%) 22
Serious TEAEs (SAEs)		n.a.	0 (0) 0	0 (0) 0	0 (0) 0	0 (0) 0
TEAEs leading to dose withdrawal		n.a.	0 (0) 0	0 (0) 0	1 (1.8%) 0	1 (1.8%) 1

**Table 3:** Distribution of adverse events by treatment arm. WA-ref: weight adjusted reference group (200 μg/Kg), FD18: fixed-dose 18 mg, FD36: fixed-dose 36 mg).

Adverse event	WA-ref (n = 54)		FD18 n = 55		FD36 n = 57		Total (n = 166)**	
	n*	%	n*	%	n*	%	n	%
Headache	5	9.3	2	3.6	3	5.3	10	6.02
Dizziness	1	1.9	-	-	-	-	1	0.60
Dysmenorrhea	1	1.9	3	5.5	1	1.8	5	5.54
Vaginal candidiasis	-	-	1	1.8	-	-	1	0.60
Throat pain	-	-	1	1.8	2	3.5	3	1.80
Cold	-	-	1	1.8	2	3.5	3	1.80
Gastroenteritis	-	-	1	1.8	-	-	1	0.60
Nausea	-	-	-	-	1	1.8	1	0.60
Tooth pain	-	-	-	-	1	1.8	1	0.60
Back pain	1	1.9	-	-	1	1.8	2	1.20
Abdominal pain	1	1.9	-	-	-	-	1	0.60
Diarrhea	2	3.7		-	1	1.8	3	1.80
Prolongued coagulation time	-		-	-	1	1.8	1	0.60

*% calculated regarding to total subjects under each treatment

**n resulting by the addition of number of subjects under each treatment

### Pharmacokinetics

The initial analysis of plasma concentrations for IVM showed that one participant presented baseline levels of IVMB1a above 5% of C_max_ in period 2, 4 participants presented baseline levels above 5% of C_max_ of IVMB1b in period 2 and 2 participants presented baseline levels above 5% of C_max_ of IVMB1b in period 3. A sensitivity analysis was conducted to evaluate if the absence of these 5 subjects with the pre-dose value greater than 5% of C_max_ value had implications in the main PK parameters. Since the results were not significantly altered, we present the main results of the study including all 54 participants.

**Fig 4:**
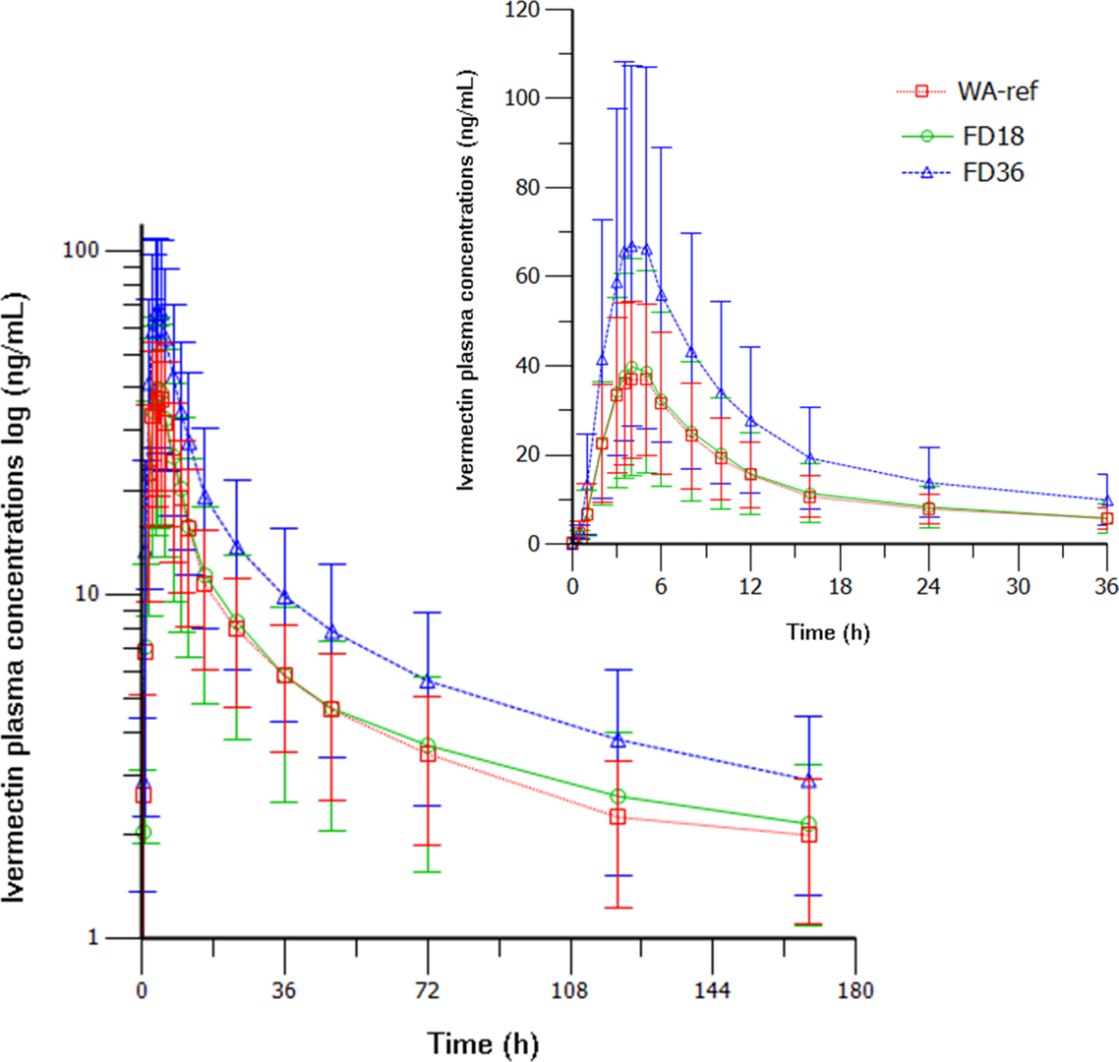
Mean IVM plasma concentration profiles in 54 individuals exposed sequentially in random order to the 3 treatment arms. Insert describe details of the time points within the initial 36 hours. WA-ref: weight adjusted reference group (200 μg/kg), FD18: fixed-dose 18 mg, FD36: fixed-dose 36 mg).

The pharmacokinetic parameters of IVM in the three study groups are shown in Table 4. Fig 4 shows the mean IVM plasma concentrations in the three treatment arms.

**Table 4:** PK parameters of IVM for entire study population and by the 3 weight strata. WA-ref: weight adjunted reference group (200 μg/kg), FD18: fixed-dose 18 mg, FD36: fixed-dose 36 mg).

Parameters (raw data)	WA-ref	FD18	FD36
	*Arithmetic mean (SD)* *Median [min- max]*	*Arithmetic mean (SD)* *Median [min- max]*	*Arithmetic mean (SD)* *Median [min- max]*
**All study groups (n = 54)**			
**AUC**_**0**_^**t**^ **(ng·h/mL)**	860.13 (384.13) 830.09 [247.01–1795.24]	885.92 (514.23) 792.60 [154.49–2340.65]	1500.40 (838.40) 1440.83 [353.68–3908.27]
**AUC**_**0**_^**∞**^ **(ng·h/mL)**	1087.81 (505.16) 1013.01 [323.20–2245.68]	1132.50 (684.05) 1057.84 [179.36–3065.69]	1906.68 (1136.50) 1629.30 [410.34–5487.41]
**C**_**max**_ **(ng/mL)**	43.19 (18.11) 44.52 [22.28–77.22]	45.23 (24.29) 38.09 [11.17–103.48]	78.04 (43.11) 65.49 [20.91–199.97]
**t**_**max**_	4.15 (1.20) 4.00 [2.00–8.00]	4.33 (1.94) 4.00 [2.00–16.00]	4.11 (1.07) 4.00 [2.00–8.00]
**t**_**1/2**_ **(h)**	80.66 (33.33) 76.73 [24.90–177.28]	80.98 (48.51) 67.36 [18.04–333.76]	90.56 (58.17) 75.44 [30.37–372.02]
**V/F (L)**	1822.29 (857.21) 1592.81 [627.07–4321.88]	2266.20 (1215.22) 2012.66 [704.01–6719.59]	3000.97 (1677.28) 2747.62 [798.15–8063.24]
**Cl/F(L/h)**	17.31 (9.16) 15.29 [5.34–46.41]	24.40 (19.14) 17.02 [5.87–100.36]	27.33 (17.71) 22.10 [6.56–87.73]
**Group 1 (n = 18)**			
**AUC**_**0**_^**t**^ **(ng·h/mL)**	866.62 (435.52) 834.27 [293.95–1795.24]	1112.80 (547.60) 1077.67 [393.25–2340.65]	1787.67 (930.102) 1625.34 [353.68–3387.34]
**AUC**_**0**_^**∞**^ **(ng·h/mL)**	1040.85 (521.02) 980.15 [353.27–2245.68]	1322.69 (707.92) 1265.90 [495.75–3065.69]	2230.69 (1362.14) 1857.52 [410.34–5487.41]
**C**_**max**_ **(ng/mL)**	44.76 (19.47) 45.33 [16.12–86.42]	56.09 (24.73) 52.19 [21.62–103.48]	96.20 (52.11) 71.80 [14.16–99.99]
**t**_**max**_	4.08 (0.96) 4.00 [2.00–6.00]	4.08 (1.30) 3.50 [2.00–8.00]	4.19 (0.94) 4.00 [3.00–6.00]
**t**_**1/2**_ **(h)**	68.18 (34.43) 59.15 [24.90–177.28]	66.85 (22.23) 62.77 [28.00–105.46]	84.63 (75.13) 65.55 [30.37–372.02]
**V/F (L)**	1272.55 (595.04) 1209.17 [627.07–2967.48]	1581.69 (818.77) 1317.25 [704.01–3528.47]	2277.92 (1129.88) 2356.62 [798.15–3844.33]
**Cl/F(L/h)**	15.01 (8.70) 12.26 [5.34–33.97]	17.73 (9.36) 14.23 [5.87–36.31]	24.40 (19.06) 19.38 [6.56–87.73]
**Mean time above 16ng/mL (h)**	10	10	22
**Group 2 (n = 18)**			
**AUC**_**0**_^**t**^ **(ng·h/mL)**	778.37 (333.93) 675.74 [247.01–1571.48]	711.90 (447.43) 639.94 [169.55–1851.18]	1298.22 (813.86) 1341.78 [499.78–3908.27]
**AUC**_**0**_^**∞**^ **(ng·h/mL)**	963.30 (401.19) 878.59 [323.20–1842.72]	890.81 (530.21) 806.42 [223.42–2298.88]	1656.78 (1053.15) 1609.95 [569.29–4655.96]
**C**_**max**_ **(ng/mL)**	38.18 (16.68) 39.34 [13.86–73.59]	37.94 (22.55) 31.72 [11.17–90.81]	65.39 (34.53) 65.49 [21.97–144.45]
**t**_**max**_	4.25 (1.57) 4.00 [3.00–8.00]	4.17 (1.21) 3.75 [3.00–8.00]	3.64 (0.89) 3.50 [2.00–5.00]
**t**_**1/2**_ **(h)**	77.46 (26.78) 76.73 [35.46–126.80]	75.32 (30.56) 64.49 [31.21–131.04]	91.77 (56.18) 79.47 [32.43–271.77]
**V/F (L)**	1978.44 (890.34) 2051.55 [814.86–3963.29]	2712.47 (1383.54) 2403.01 [1011.58–6719.59]	3488.51 (1846.32) 3230.07 [837.49–7101.50]
**Cl/F(L/h)**	18.80 (9.73) 17.08 [8.14–46.41]	29.44 (20.53) 22.44 [7.83–80.57]	30.73 (17.71) 22.36 [7.73–63.24]
**Mean time above 16ng/mL (h)**	8	8	14
**Group 3 (n = 18)**			
**AUC**_**0**_^**t**^ **(ng·h/mL)**	935.41 (381.72) 862.69 [376.98–1641.39]	833.05 (484.58) 792.60 [154.49–1706.92]	1415.33 (725.01) 1456.71 [433.14–2858.74]
**AUC**_**0**_^**∞**^ **(ng·h/mL)**	1259.27 (559.71) 1153.64 [524.27–2235.71]	1184.00 (757.37) 1128.59 [179.36–2606.35]	1832.58 (937.65) 1677.87 [532.32–3296.75]
**C**_**max**_ **(ng/mL)**	46.64 (17.98) 44.52 [22.28–80.61]	41.65 (22.91) 37.08 [12.54–101.36]	72.52 (36.54) 75.16 [20.91–132.46]
**t**_**max**_	4.11 (1.05) 3.75 [3.00–6.00]	4.75 (2.98) 4.00 [3.00–16.00]	4.5 (1.21) 4.50 [3.00–8.00]
**t**_**1/2**_ **(h)**	96.33 (33.55) 100.47 [31.54–148.23]	100.78 (72.43) 77.67 [18.04–333.76]	95.30 (41.05) 97.15 [36.35–185.58]
**V/F (L)**	2215.89 (796.42) 2065.75 [1346.79–4321.88]	2504.44 (1119.11) 2438.91 [847.94–4705.04]	3236.49 (1794.78) 2790.83 [1119.26–8063.24]
**Cl/F(L/h)**	18.12 (9.09) 16.14 [8.25–34.33]	26.04 (23.57) 15.97 [6.91–100.36]	26.86 (16.72) 21.54 [10.92–67.63]
**Mean time above 16ng/mL (h)**	10	8	14

The parameters related with drug exposure (AUC_0_^t^ and C_max_) showed a high interindividual coefficient of variation (CV) (CV = 37.4% and CV = 32.5%) and intraindividual variability (CV = 39.6% and CV = 33.2%) respectively. When comparing the systemic bioavailability (AUC_0_^t^ and C_max_) of WA-ref with the other two study groups using fixed doses, we observed an overall increase in AUC_0_^t^ of 2.9% for FD18 and a 74.44% increase for FD36. These higher values were observed in C_max_ as well, showing a similar increase of 4.7% of the systemic bioavailability for FD18 and a 80.69% increase for FD36. The analysis of the relationship (ANCOVA) between IVM PK parameters with BMI and weight indicates that individuals with high BMI and weight present higher V/F and t_1/2_. However, no significant association was found between weight and BMI with Cmax and AUC_0_^t^ (Table 5).

**Table 5:** Effect of BMI and weight in PK parameters.

Parameters All study groups (n = 54)	BMI (p-value)	Weight (p-value)
t_1/2_	<0.001	0.001
V/F	<0.001	0.001
Cl/F	0.902	0.461
C_max_	0.365	0.115
AUC_0_^t^	0.501	0.288

ANCOVA with BMI and Weight as covariates.

The median time (hours) which the participants presented plasma IVM levels above those described as the lethal concentration 50 (LC_50_) against *Anopheles gambiae s*.*s*. (>16 ng/ml) was 8 h for WA-ref, 8 h for FD18 and 14 h for FD36 (p<0.001).

## Discussion

We report in this study safety and pharmacokinetic results of an alternative dosing regimen for IVM. These results are of particular relevance for public health interventions based on preventive chemotherapy through MDA of anthelmintics as those used for onchocerciasis and LF and under study for other NTDs like STH and scabies, as well as malaria [9, 30, 31]. This study provides the first pharmacokinetic and safety data of a formulation of IVM in 18 mg tablets, which adds further logistical advantages for fixed dosing as proposed in this study and sets the pharmaceutical conditions for an eventual co-formulation with other anthelmintics like albendazole for the control of LF, *Trichuris trichiura* and *S*. *stercoralis* in areas where these species are found among the prevalent STH.

Safety data was consistent with previous studies regarding the lack of significant adverse events even at the highest doses uses in this study (36 mg) which in the lowest weight group (51 to 65 kg) providing doses of up to 700 mcg/kg [16, 32, 33]. Changes in Hb observed through the study, even in the control group treated with usual dosing, might be explained by the frequent blood draws although further studies might be needed. However, these hematologic results were not found in other smaller studies using IVM at doses up to 2000 mcg/kg in healthy volunteers or 800 mcg/kg in individuals infected with *O*. *volvulus* [16, 34]. Although IVM was very well tolerated, 14 participants received treatment to control adverse events that were mostly to improve mild headache, common in participants of phase I trials after deprivation of caffeine and other substances. At the same time that fixed and higher doses of IVM proved to have an excellent safety profile in our study, higher systemic exposure (AUC_0_^t^ increased approximately 65% for FD36) of plasma IVM were achieved overall among study participants. Although no efficacy evaluation has been done, similar or higher efficacy is expected against the common pathogens targeted by IVM, while keeping a good safety profile and facilitating delivery of the drug. As expected, higher levels of AUC_0_^t^ and C_max_ were found in participants of group 1, compared to the heavier group of participants in group 3 (FD36). These differences in systemic exposure among participants having different weights might have implications on the efficacy of ivermectin, potentially achieving higher cure rates in those patients with lower weight.

Values of AUC_0_^t^ and C_max_ at the three dose studied are consistent with linear behavior previously reported by Guzzo et al. with single doses of up to 120 mg [16]. Pharmacokinetic parameters related with drug exposure in magnitude (AUC_0_^t^, AUC_0_^∞^) and rate (C_max_) showed a high inter and intra individual variability, as reported by other authors [15, 16, 24, 35]. The absorption parameter t_max_ is comparable to that reported in other studies in healthy volunteers [15, 16, 35]. Disposition parameters (V/F and Cl/F) are rarely reported in IVM pharmacokinetic studies in healthy volunteers. However, the values obtained in our study for Cl are in the range from what has been previously reported from 12 to 30 L/h [36][37].

It has been recently suggested that an increased drug variability associated with suboptimal drug concentrations may have implications on the development of IVM resistance [11]. However, this data is based in modeling studies in veterinary medicine, and still more studies are needed to confirm this hypothesis. The strategy proposed in this study is not only based in a fixed-doses of IVM but also is based in the use of a high dose, ranging from 200 mcg/kg for those weighting 90 kg to 360 mcg/kg for those weighting 50 kg. Thus, we ensure that patients do not receive lower doses of the drug, and most of them receive a superior dose of IVM.

Most notably, elimination half life was long enough to still be detectable at significant levels (5% of the C_max_) after the 14-day wash-out period in 5 cases, which although proved not to affect the analysis, highlights the persistence of IVM in plasma in some participants. The elimination half life ranged between 60 to 100 hours in the different weight groups, with increasing values in the individuals with higher BMI and weight probably reflecting the high liposolubility of IVM with longer retention times proportional to the presence of more adipose tissue; an explanation also consistent with the finding of longer half lifes in females than in males reported by other authors [38][14]. Our findings reveal longer half lifes of IVM than other studies reporting values from 12 to 28 h in healthy volunteers, [36] [16][23] However, this could be expIained due to the fact that other authors reported studies with shorter follow-up periods and detectable IVM levels at the latest timepoints (56 and 60 hours) [36][16]

The use of IVM for the different indications for which it has a demonstrated clinical usefulness has dosing strategies that in all instances are based on weight based dosing. Although dose finding experiments have identified the appropriate dosing like in onchocerciasis, where doses higher than 150 mcg/kg appear to have no increments in efficacy (neither toxicity) [32], there are no target plasma drug levels or adequate markers of efficacy. A potential exception is being attempted on the benefits of IVM as a mosquitocidal drug for the control of malaria, where a lethal concentration 50 (LC50) against *Anopheles gambiae s*.*s*. has been estimated at 15.9 ng/ml [13]. In our study, those concentrations were maintained for 8 hours in the WA-ref and FD18 and for 14 hours in the FD36 group. Strategies are currently being evaluated to mantain IVM concentrations in human blood at mosquitocidal levels [39]. Our proposal of using high and fixed dose of IVM could be helpful to prolong IVM concentrations at levels that could have impact on *Anopheles* mosquitoes, although probably combined with other strategies, such as increasing the drug administration to multiple-days regimens [40]

The limitations of this study include the healthy, non-infected status of the volunteers; although this limitation might not be relevant based on a previous study showing no differences in PK parameters between *O*. *volvulus* infected individuals and controls [35]. Whether the same applies for individuals infected with gut-dwelling parasites is currently unknown. Another limitation is the use of a different IVM for the reference group rather than the widely used Mectizan donated by Merck, which is used in the large majority of MDA programs. However, the Abbott labs IVM used in this study is the reference IVM product in Brazil [23].

In conclusion, the administration of IVM in a fixed dosing strategy with 18 mg or 36 mg is as safe as the reference product adjusted by weight, adding a potential benefit due to the increased systemic exposure to the drug particularly in low weight adult individuals. Moreover, the fixed dose regimen offers a logistical advantage for the deployment of large MDA interventions aiming at the control and interruption of transmission of NTDs, and facilitates the co-administration with other antihelmintics prescribed this way, like albendazole or mebendazole. Further studies evaluating these concepts in pediatric populations and infected individuals, as well as clinical trials with efficacy endpoints are warranted.
